# The Role of Herbal and Nutritional Treatments in the Fight against COVID-19 and Other Respiratory Tract Infections

**DOI:** 10.3390/ijerph182212001

**Published:** 2021-11-16

**Authors:** Aline El Zakhem, May Annie Chalhoub, Maya Bassil

**Affiliations:** 1 Division of Infectious Diseases, American University of Beirut Medical Center, Beirut 1107, Lebanon; az51@aub.edu.lb; 2 Department of Natural Sciences, Lebanese American University, Beirut 1102, Lebanon; mayannie.chalhoub@lau.edu

**Keywords:** coronavirus, COVID-19, respiratory infections, Chinese herbal medicine, nutrition, micronutrients

## Abstract

With the growing spread of COVID-19 worldwide, the appeal to alternative and nutritional therapies in conjunction with medical therapies has been heightened. This article aims to review studies assessing the roles of Chinese traditional medicine and nutrition in upper respiratory infections, including COVID-19. Various Chinese herbal protocols have been shown to fight respiratory infections, with several having been tested on the novel coronavirus. Additionally, promising findings have been reported when medical treatments were complemented with nutritional interventions. Supplementation with vitamins C and D, Zinc and Selenium are discussed, in addition to certain phytochemicals and food that also possess immunoregulatory and antiviral properties. Further clinical studies are needed to establish these alternative treatments as part of the management of emerging respiratory infections.

## 1. Introduction

In December 2019, in Wuhan China, the first human cases were detected for a novel acute infectious respiratory disease that primarily attacks the respiratory tract. The new coronavirus disease (COVID-19) shares similar clinical presentations with other coronavirus infections, mainly Severe Acute Respiratory Syndrome (SARS) and Middle East Respiratory Syndrome (MERS) [1,2]. On 11 March 2020, the World Health Organization (WHO) announced that COVID-19 was a pandemic that had spread to over 110 countries within the course of months. Patients with co-morbidities and older adults are more prone for severe manifestations of the disease. COVID-19 has symptoms similar to those of contracting a mild flu (fever, cough, and malaise), although asymptomatic cases and other atypical symptoms have been reported as well [3,4]. In severe cases, symptoms can rapidly develop and lead to respiratory failure and even multi-organ failure, necessitating admission to the intensive care unit (ICU) and initiating mechanical ventilation [5,6]. In 2020, the number of deaths reported to WHO as a result of COVID-19 was estimated to have exceeded 3.3 million deaths. To date, septic shock and organ failure secondary to severe pulmonary infection from COVID-19 are the main causes of death [5].

Growing evidence supports the use of many therapeutics for the management of COVID-19 infections, depending on the severity of the disease. These include antivirals, targeted monoclonal antibodies, and immunomodulators [7]. However, the management protocol depends mainly on the availability of these drugs in the region as well as the approval of related authorities. In parallel, and with the growing spread of COVID-19 worldwide, it has become increasingly common to resort to alternative therapies, including the use of herbal and nutritional supplements. Alternative medicine, including Chinese herbal medicine, has existed for centuries, and appears to be promising for the treatment of acute respiratory infections [8]^.^ Recent trials have shed further light on the roles of such herbs in the treatment of COVID-19 [9]. Moreover, several nutritional treatments have also aided in the treatment of respiratory viral infections. This review aims to discuss studies investigating herbal and nutritional treatments of COVID-19 and other respiratory tract infections. 

## 2. Methodology 

Five electronic databases (PubMed, Cochrane Library, ScienceDirect, Google Scholar, JAMA) were searched with the following search items: traditional Chinese medicine, Chinese herbal medicine, alternative therapy, COVID-19, SARS, SARS-CoV-2, randomized clinical trial, vitamin C, vitamin D, zinc, nutrition, curcumin, flavonoids, selenium, zinc, ARI, ARDS. Clinical trials, observational studies, and reviews were included. Only studies related to upper respiratory tract infections dated 2010 and later were included. Preliminary studies on SARS were excluded.

## 3. Chinese Herbal Medicine

Chinese herbal medicine (CHM) has been adopted in China for centuries as an alternative medicine, whereby plants are used to alleviate symptoms or treat diseases. CHM is a complex concept guided by the type of herb and the patient’s signs and symptoms [9]. Several studies have identified the potency of CHM as a source of antiviral compounds in fighting SARS. A meta-analysis reviewed four herbal extracts: *Lycoris radiata*, *Artemisis annua*, *Pyrsoia lingua*, and *Lindera aggregata* that had been shown to significantly inhibit progression of SARS in a dose-dependent manner by blocking the virus from replicating and remaining viable. All four compounds proved to be more effective than interferon alpha (INF-α), an immunotherapy drug. The best potency, however, was detected for the alkaloid bulb of *Lycoris radiata*, Lycorine [8]. Lycorine’s mode of action is still vague; however, unlike antiviral drugs that directly target viral protein and ribonucleic acid (RNA) polymerase activity [3], studies suggested that it inhibits the export of viral ribosomal proteins from the nucleus to the cytoplasm and the structural changes that coronaviruses induce in host cells [10].

Yupingfeng powder (YP) (including Astragali Radix, Glycyrrhizae Radix Et Rhizoma, Saposhnikoviae Radix, Atractylodis Macrocephalae Rhizoma, Lonicerae Japonicae Flos, and Forsythiae Fructus) showed prophylactic effects against SARS in hospital workers. When given, none of the participants in the trial caught SARS. YP is an antiviral that improves and maintains the mucosal integrity of the upper respiratory tract [3].

Furthermore, Glycyrrhizin, an extract from liquorice root, has demonstrated inhibitory potentials on the replication of the SARS by interfering in cellular signaling pathways; protein kinase and casein kinase II, and transcription factors. It also inhibits viral replication by inducing nitric oxide synthase, thus elevating levels of nitric oxide [11]. In rat models, diets supplemented with 150 mg/kg/day Glycyrrhizin compared to placebo showed a reduction in the expression of ACE2 protein and gene, therefore reducing the uptake of COVID-19 at the cellular level and its spread. A significant main effect was detected in the MRNA ACE2 coding at the level of the small intestine (*p* = 0.04) [12].

Combining herbal therapies with conventional medicine showed synergistic beneficial results in combatting SARS. Conventional medicine resorts only to pharmacological antiviral and antibacterial drugs and immunomodulators, whereas combination medicine includes both pharmacological and herbal therapies. A pool of five randomized clinical trials (RCTs) suggest that combination medication may have more health benefits compared to conventional medicine alone, although methodological limitations render these findings inconclusive [8].

A total of 26 herbs that are used as a course of treatment in viral respiratory tract infections were identified as containing 13 compounds that have been suggested to have binding capacity to SARS-CoV and SARS-CoV-2 [9].

Provinces in China have taken individual initiative in releasing statements related to herbal treatments. All provinces used the six herbs in YP for tonification and protection. The top two herbs used were *Radix astragali* and *Glycyrrhizae Radix Et Rhizoma* [13]. Both are associated with having qi blood, a tonifying effect that replenishes the body when weak [12]. Individual case studies also tested the effectiveness of *Shuanghuanglian* oral liquid (SHL) (including *honeysuckle*, *forsythia*, and *Scutellaria baicalensis*). Two patients had a clinical manifestation of COVID-19 with patches on the lungs, but tested negative for the virus. When isolated and given SHL at a rate of 20 mL twice per day to three times per day, the patients exhibited a drop in body temperature and reduction in cough intensity three days later with significant improvements in their lung computer tomography (CT) scans one week later [13]. SHL’s historical usage is to treat sore throat, cough, and fever (similar clinical manifestations of COVID-19) [14].

Additionally, Lianhuaqingwen (LH) capsules combining different herbs have been found to alleviate symptoms and shorten the course of COVID-19. LH is made from *Forsythia suspensa*, *Lonicera japonica*, *Ephedra sinica*, *Isatis indigotica*, *Pogostemon cablin*, *Rheum palmatum*, *Glycyrrhiza uralensis*, *Dryopteris crassirhizoma*, *Rhodiola crenulata*, *Houttuynia cordata*, *Prunus sibirica*, *gypsum* and *1-menthol*. A RCT among COVID-19 patients from 23 hospitals in China randomized patients to either an antiviral pharmacological treatment or a combination of pharmacological treatment and LH capsules (four capsules thrice daily for 14 days) [15]. After 14 days, the combination group showed a faster recovery from symptoms compared to the pharmacological group (*p* = 0.022), and shorter median time to symptom recovery (7 days in combination vs. 10 days in control). However, no difference in viral concentrations was detected between the two groups [15]. LH works through the suppression of viral loads in the cytoplasm and cellular membrane and replication of COVID-19 and H1N1 and can inhibit the release of tumor necrosis factor-alpha (TNF-α), interleukin-6 (IL-6), and macrophages, all of which are components in the cytokine storm. LH components prevent the binding of COVID-19 with the angiotensin-converting enzyme (ACE), antagonize the binding of spike protein and ACE and suppress inflammatory mediators release, improve the host-defense of the gastrointestinal tract, and preserve lung integrity via the suppression of oxidative stress and apoptosis and pulmonary inflammation [15].

A meta-analysis reported better results among patients receiving CHM combined with medications, compared to medications alone. Pooled scores of signs and symptoms showed that among the combined CHM and medications group, scores were 1.3 lower compared to medication group alone, despite the 94% heterogeneity due to different scoring methods. Participants in the combination group showed more favorable improvements in their lung CT scans (RR 1.34, 95% CI [1.19, 1.51]) [16]. Consistently, in a retrospective cohort on critical COVID-19 patients in Wuhan, patients receiving CHM had an 82% lower mortality risk compared to the non-users group (odds ratio 0.178, 95% CI 0.076–0.418; *p* < 0.001) [15]. Additionally, *Qingfei paidu* was able to aid in the treatment of 98% of COVID-19 cases in Sichuan province, with 41% recovery and 26% significant efficacy within nine days [17]. Completed and Planned Clinical Trials on CHM in patients with COVID-19 are presented in Table 1.

In a review article on CHM, the usage of the herbs was categorized on the basis of the phases of infection. During the early phase of infection, *Ma xing shi gan* (including *Ephe-dra*, *Apricot Kernel*, *Gypsum*, and *Licorice Decoction)* is recommended to treat cough and asthma and gastric complications, while *Gancao ganjiang* (including *Glycyrrhiza uralensis Fisch*. And *Zingiber officinale*) targets chest and back pains, and dizziness. During the infection phase, *Sheganmahuang* formula (including *Asarum sieboldii Miq.*, *Aster tataricus L.f.*, *Ephedra sinica Stapf*, *Belamcanda chinensis (L.) Redouté*, *Pinellia ternate (Thunb.) Breit.*, *Schisandra chinensis (Turcz.) Baill.*, *Tussilago farfara L.*, *Zingiber officinale Roscoe*, and *Ziziphus jujuba Mill*) is useful in fighting asthma, *Maxingshigan* reduces lung inflammation by lowering inflammatory markers, LH acts as an anti-inflammatory and relieves coughs and phlegm. Lastly, during the recovery phase, *Shengmai San* (including *Radix ginseng*, *Radix ophiopogonis*, and *Fructus schisandrae*) have been shown to improve blood circulation, cardiac function, and overall lung integrity [23].

It is worth noting that the use of CHM is also associated with potential adverse effects including diarrhea, sore throat, and nausea [17]. Resorting to medicinal plants can even lead to unintended intoxications, whereby some plants are contaminated with microorganisms, fungal toxins, and even pesticides and heavy metals. Unsupervised intake of herbal medications can also interfere with mode of action of conventional medications [24].

## 4. Nutritional Therapies and Supplements

Over the past decade, the dietary supplement market has witnessed a hike in sales. Still, several weeks prior to the first COVID-19 wave, sales increased by 44% compared to 5% in the previous year in the United States. Similar trends were detected in the United Kingdom and France, where sales increased by 63% and 40%, respectively. This shift in market demand is attributed to the allegedly “immune-boosting” effects of vitamins and minerals [25]. 

### 4.1. Vitamin D

Vitamin D has commonly been found to be inversely associated with the risk of developing acute respiratory viral infections (ARI), rendering it integral in the fight against COVID-19 [26,27]. A recent review of clinical trials revealed significant associations between in vivo vitamin D supplementation and risk of developing ARI, despite conflicting results in few reports. All studies supplemented their participants with a daily dose 1500 IU of oral cholecalciferol. Interestingly, better clinical results were obtained when vitamin D was administered weekly or daily rather than as a one-time bolus monthly or every three months. Bolus supplementation might dysregulate enzymes responsible for synthesis and degradation of 1,25-dihydroxyvitamin D, thus decreasing its concentrations in post-renal tissues [27]. One RCT showed that the supplementation had a protective effect on participants who had baseline serum 25-Hydroxyviatmin D (25(OH)D) below 10 ng/mL by 36% (OR 1.36 95%CI 1.01–1.84), while another showed a 7% risk decrease in self-reported ARI with each 4 ng/mL increase in serum 25(OH)D. Subgroup analysis furthermore indicated that the effect was only significant among participants with serum 25(OH)D below 25 nmol/L (*p* = 0.002) [28,29]. A systematic review and meta-analysis of RCTs showed that vitamin D supplementation was a safe protective agent against ARI [30].

Emerging epidemiological findings link serum 25(OH)D concentrations to COVID-19 disease incidence or prevalence [27]. Two national European investigations showed that lower circulating 25(OH)D was associated with a higher COVID-19 severity [31]. D’avolio et al. revealed that patients that tested positive for COVID-19 had a median 25(OH)D of 11.1 ng/dL, while that of individuals who tested negative was 24.6 ng/mL (*p* < 0.004) [31]. Similarly, an observational study in the United Arab Emirates showed that vitamin D levels <12 ng/mL upon hospital admission were associated with a more severe COVID-19 infection (*p* = 0.005) after adjusting for risk factors (age, sex, smoking, and comorbidities). As for the death risk, after adjusting for age and sex, admitted patients with serum 25 (OH)D <12 ng/mL had a 2.55 times higher risk of death (*p* = 0.04), which increased to 2.58 times (*p* = 0.048) when comorbidities were entered into the model [32].

Additionally, the role of ethnicity in affecting the relationship between vitamin D status and the likelihood of testing positive for COVID-19 was studied retrospectively by Meltzer et al. Findings revealed that black individuals were 2.64 times more likely to test positive if they had vitamin D levels of 30 ng/mL to 40 ng/mL, while no association was found among white participants [28].

Supplementing COVID-19 patients with Vitamin D presents several challenges. Hospitalized patients are already in a hyperinflammatory state, and the effects of micronutrient supplementations might be masked in the presence of medications. Another study on COVID-19 cases examined the association between the serum 25(OH)D levels and clinical symptoms. Cases were classified as: (1) mild–mild clinical features without pneumonia diagnosis, (2) ordinary–confirmed pneumonia with fever and other respiratory symptoms, (3) severe–hypoxia (at most 93% oxygen saturation) and respiratory distress, and (4) critical–respiratory failure. Serum 25(OH)D levels were inversely associated with the severity of clinical outcomes (*p* < 0.001). The odds of having mild outcomes rather than ordinary were 1.63 times for each increase in standard deviation (SD) of 25(OH)D (*p* = 0.007). Additionally, for each 1 SD increase in serum 25(OH)D, the odds of having mild outcomes rather that severe was 7.94 times higher (*p* < 0.001), while the odds of having mild outcomes rather than critical ones was 19.61 times higher (*p* < 0.001) [27]. A systematic review and meta-analysis of 43 observational studies found that the risk of being infected with COVID-19 is inversely related to vitamin D values (OR = 1.26; 95% CI, 1.19–1.34; *p* < 0.01). Comparing vitamin D deficient patients to non-deficient, the severity of the disease and mortality risk were significantly higher (OR = 2.6; 95% CI, 1.84–3.67; *p* < 0.01 and OR = 1.22; 95% CI, 1.04–1.43; *p* < 0.01, respectively) [29]. In a double masked RCT, COVID-19 patients on a combination of HCQ (400 mg every 12 h on day one then 200 mg for the following five days) and azithromycin (500 mg orally for five days) were allocated to either no calcifediol or an oral calcifediol of 0.532 mg upon admission. Patients in the intervention were given 0.266 mg of calcifediol on days three, seven, then weekly till discharge. Results showed that including calcifediol in the treatment reduced the need for ICU transfer (OR: 0.03; 95%CI: 0.003–0.25). Among the patients in the intervention group, none died, and all were discharged with no recorded complications [33]. Conversely, a randomized clinical trial conducted on 240 hospitalized patients with moderate to severe COVID-19 reported no improvement in hospital stay following a single dose of 200,000 IU of vitamin [34]. The latest Cochrane review revealed that to date, not enough good-quality evidence has been found on the use of vitamin D as safe and effective treatment in the fight against COVID-19 [34]. Discrepancies in the findings so far could be related to differences in the routes and forms of vitamin D supplementation and in the heterogeneity in subject recruitment in terms of severity of the disease and preexisting (or lack thereof) of diagnosed vitamin D deficiency [35]. A recent multicenter RCT showed that a daily 5000 IU dose of vitamin D for two weeks significantly increased serum 25 (OH) D (*p* = 0.003), and reduced time to recovery (*p* = 0.039) and aguesia (*p* = 0.0035) [36].

Vitamin D boosts the body in the fight against viral infections in three domains: physical barrier, natural immunity, and adaptive immunity. At the cellular level, the active form of vitamin D preserves the junction integrity between cells that are highly compromised during viral infections. It also decreases the damage inflicted on cells due to the induced cytokine storm and decreases pro-inflammatory cytokines, including TNF and INF gamma (INF-γ), while enhancing the expression of anti-inflammatory cytokines as a result of macrophages. It also enhances innate immunity by releasing antimicrobial peptides; cathelicidin and defensins. Cathelicidin directly attacks enveloped and non-enveloped viruses by disturbing viral cell membranes and neutralizing the biological activity of their endotoxins. With respect to adaptive immunity, the active form of vitamin D suppresses T-1 helper cells, leading to the release of inflammatory cytokines and INF-γ, and enhances the release of T-2 helper cells that inhibit the release of T-1 cells. This induces the release of T regulatory cells that stop any inflammation release. High levels of vitamin D also increase genetic expressions that improve body anti-oxidative capacity and increase the levels of glutathione, sparing vitamin C usage, which is another potent antioxidant [28,31,37,38].

### 4.2. Vitamin C

Clinical studies have shown the effectiveness of vitamin C as an antiviral treatment in acute respiratory disease syndrome (ARDS). During the SARS outbreak, vitamin C was widely consumed as a preventative measure, and participants who took vitamin C supplements were asymptomatic. A RCT on older adults showed that 200 mg/day of vitamin C improved respiratory symptoms among severely ill patients and recorded 80% fewer deaths when compared to placebo [38]. In a case report by Cheng (2020) [39], a patient with ARDS was given a dose of 200 mg/kg body weight of vitamin C daily. Significant progress was found in X-ray imaging within 2 days, and 2 months later the patient was cured [39].

The countries with the highest COVID-19 cases are low- to middle-income countries known to have high rates of hypovitaminosis C, indicating that vitamin C deficiency can overlap with COVID-19 risk factors [40]. Intravenous (IV) pharmacological administration of vitamin C (200 mg/kg body weight/day) divided into four doses in COVID-19 patients, resulted in a 97.8% reduction in ICU stay. Additionally, a drop-in mortality rate was detected reaching 8.5% in the treatment group and 40.4% in the control group (*p* < 0.001) [39]. Additionally, when IV vitamin C was administered to 50 COVID-19 patients with moderate to severe disease intensity, at a dose of 2 to 10 g/day over eight to ten hours, oxygenation index was improved, and all patients were eventually discharged [39]. Another study on COVID-19 patients showed that one g of IV vitamin C for three days caused a significant drop in inflammatory markers [40]. In the United States (US), ICU patients on a 1500 mg regimen of vitamin C (four times per day) showed significant improvements compared with patients not receiving supplementations [41]. Another cohort study on 76 COVID-19 patients stratified them to either receive 6 g vitamin C every 12 h on the first day and 6 g once a day for the next four days or standard therapy. Participants receiving vitamin C had a reduced 28-day mortality risk (HR = 0.14, 95% CI, 0.03–0.72) and improved oxygen status (63.9% for vitamin C group versus 36.1% for the standard therapy group) [42]. Among ICU-admitted COVID-19 patients, the majority had hypovitaminosis C (mean level below 22 µmol/L). The mean level of vitamin C for survivors was 29 µmol/L, while levels were 15 µmol/L for non survivors [43]. Consistently, a study in New Zealand showed that pneumonic COVID-19 patients had significantly lower vitamin C levels compared to healthy patients (*p* < 0.001) [43]. However, not all studies found a significant effect of vitamin C on COVID-19. A randomized open label clinical trial randomized participants to either receive ritonavir and HCQ or high dose of intravenous vitamin C (6 g per day) with the same regimen. The length of hospitalization among participants receiving vitamin C was significantly longer than those on the traditional regimen (8.5 days versus 6.5 days respectively) (*p* = 0.028). Additionally, upon discharge, no significant difference between clinical outcomes (length of stay, mortality, and oxygen saturation) was detected [44].

ARDS is accompanied by high oxidative stress due to excessive release of free radicals and cytokines that will ultimately result in cellular and organ damage, in addition to lung capillary endothelial cell activation and neutrophil infiltration. It also leads to a state of hypoxia and damage to the alveoli. Being a potent antioxidant, vitamin C shows protective mechanisms against these injuries. Furthermore, in pharmacological doses, vitamin C becomes a pro-oxidant, enhancing the release the hydrogen peroxide which targets viruses by improving chemotaxis. Vitamin C can also promote phagocyte progression, oxidative death, and lymphocyte proliferation, all of which render vitamin C a potential defense in the management of COVID-19 [45]. Severe cases of COVID-19 can lead to endothelial damage that can deteriorate the health status of patients. Vitamin C can reduce the risk of complications by restoring cells’ endothelial functions [43].

### 4.3. Zinc

Zinc (Zn) is known to be a potent antiviral, antibacterial, and immunoregulatory micronutrient [45]. Zn is supplemented as part of the treatment of coronavirus [29], particularly in the most common protocols using chloroquine (CQ) or hydroxychloroquine (HCQ) with azithromycin. Indeed, a large study on patients with COVID-19-like symptoms included 220 mg Zn sulfate once daily in addition to HCQ 200 mg twice daily and azithromycin 500 mg once daily, for a total of 5 days [46]. High intracellular Zn was shown to increase the efficiency of CQ and HCQ on the inhibition of RNA-dependent RNA polymerase (RdRp), which is an essential protein encoded in the genomes of RNA viruses [46]. CQ and HCQ act as weak bases that disrupt the cellular signaling of lysosomes and Golgi. They work by increasing lysosomal pH; thus, the bioavailability of these drugs largely relies on protonization with Zn^2+^ to increase their affinity to low pH organelles [45].

However, no association between Zn intake and the risk of developing ARI was observed in a cohort study on women for Zn intakes above of 7.5 mg/day. A higher Zn intake was associated with an increased risk of developing ARI (CI 95%, 1.04–2.16) at levels above 10 mg/day among men due to Zn becoming a pro-oxidant [47].

Zn antiviral properties are mediated through its elevated intracellular concentrations. Zn modulates the structure of viral proteins and was found to directly work on the RNA of SARS by reducing its replicative abilities. Several drugs, including disulfiram, promote Zn release from papain-like protease in MERS and SARS, causing protein destabilization. In addition, COVID-19, much like SARS, needs ACE2 to enter target cells, and Zn levels of 100 µM have been shown to reduce ACE2 activity in the lungs of rats. Consistently, Zn supplementation in animals has resulted in significant improvements in lung epithelium by enhancing muco-ciliary clearance, cilia length, and tight cellular junctions (improving barrier functions) [48].

A retrospective study on hospitalized COVID-19 patients showed that patients with serum Zn levels <50 µg/dL had more severe clinical presentations and higher inflammatory markers: CRP (*p* = 0.03), IL-6 (*p* < 0.001). Having serum Zn < 50 µg/dL increased the risk of mortality by 21%, while having higher values had a 5% risk mortality (*p* < 0.001) [49].

Hospitalized COVID-19 patients receiving Zn supplements exhibited reduced hospital mortality by 24% and were discharged home sooner (*p* = 0.003). A prominent feature of COVID-19 is the loss of taste and smell attributed to the destruction of sensory cells or viral entry to the brain. Zinc deficiency has been directly associated with a reduction in the sense of taste; thus, zinc supplementation has been reported to be favorable to chemosensory abilities. Other studies, however, showed no association between Zn supplementations and olfactory dysregulations [50].

More recently, Ali et al. retrospectively assessed the role of Zinc in COVID-19 prevention and mortality among Asians and Europeans. Zinc deficiency was two times higher among Asians (17.5%), which could have led to a significant positive association between zinc deficiency and COVID-19 (*p* < 0.05), but not with mortality. Among the European population, zinc deficiency was less likely to be detected (8.9%), and a significant negative correlation was found between COVID-19 cases and death per million (*p* < 0.05) [50]. In another recent study, investigators found that COVID-19 patients who died had lower plasma zinc (43 μg/dL) compared to those who survived (63.1 μg/dL). This was reflected in an inverse relationship between plasma zinc upon admission and mortality, whereby for every unit increase in plasma zinc there was a 7% reduced risk of in hospital mortality. Plasma zinc less than 50 μg/dL at admission was associated with a 2.3-fold increased risk of mortality. In line with this, a study in Japan on 62 COVID-19 patients found that serum zinc was predictive of disease severity [49].

An RCT aimed to test possible synergistic effects of high doses of zinc and ascorbic acid on symptom length and reduction. Patients were randomized to either receive 50 mg of Zinc gluconate, 8000 mg of ascorbic acid, both nutrients, or the standard care for 10 days. No significant differences were detected among the four groups in the reduction of symptoms (*p* = 0.45). Patients in the standard remedy group had a 50% reduction in symptoms after 6.7 days, while it took 5.9 days with zinc sulfonate, 5.5 days with ascorbic acid, and 5.5 days with the combination. No significant differences were detected in the number of days for vanishing of symptoms, number of hospitalized patients, or deaths [51].

### 4.4. Other Nutrients and Phytochemicals

Selenium (Se) is a potent antioxidant. A daily 100 mcg intake of Se has shown positive effects in fighting viral infections (including SARS and H1N1) [28]. Studying Se status in different regions in China showed that in areas known to have a high Se status, higher rates of cure among victims were detected, and vice-versa [49]. A cross-sectional study in a German hospital showed that 64.7% of deceased COVID-19 patients were Se deficient, whereas only 39% of those that were Se deficient survived [51]. Se supplementation was associated with a significant reduction in viral mutations and a boosted immunocompetency in patients with known Se deficiency. Se activates T cell multiplication, thus enhancing the innate immune system and affecting thyroid hormone ratios that are a part of developing cellular immunity and cytokines [52].

A meta-analysis reported that upper respiratory tract infections (URTI) were similar in groups supplemented with flavonoids and the control group, indicating an absence of association (0.83 95%CI, 0.75–1.11). Four studies have reported that sick days were also similar between both groups (*p* = 0.16). However, supplementations with flavonoids in cohort studies significantly decreased missed workdays (*p* = 0.035) and symptoms (*p* < 0.001) compared to control [53]. Flavonoids have antiviral mechanisms; quercetin and hesperidin have anti-replicative effects, while quercetin and catechin have anti-infective effects. Flavonoids have a high binding affinity to the spike proteins and ACE2 receptors on the novel coronavirus, causing a conformational change that inhibits viral entry. Compared to CQ, flavonoids were found to be equally or even more potent in binding to the virus [53]. Different flavonoids are involved in ACE1 and ACE2 inhibition via several mechanisms [52]. Quercetin inhibits proteases, while kaempferol interacts with ACE2 via a method that inhibits viral entry. Flavonoids protect the body against ROS and their having affinity to hydrophilic amino acids aids in repairing any viral induced cell damage [51]. Moreover, vitamin C has been shown to have synergistic antiviral properties when given with quercetin [40]. Adequate flavonoids can be achieved from several good sources (100 g/serving) such as 250 mL of green tea, 100 g dark chocolate, and 100 g blueberries [54].

Curcumin is known to be an antioxidant, anti-inflammatory, and immunomodulatory natural molecule. Curcumin has shown good binding affinity with the virus’ nucleocapsid, a pro-inflammatory protein. The structure RNA of COVID-19 was determined to have a groove between the palm and finger regions at the RNA binding site, with curcumin showing a binding affinity to this region. It also might inhibit the nucleocapsid and suppress components of cellular signaling pathways that aid in infected cells growth by inhibiting protein kinases and activating enzyme cyclooxygenase [55]. The antiviral properties of curcumin stem from their ability to act in cellular signaling pathways (apoptosis and inflammation) and interfere in the viral replication cycle (genome replication and viral attachment) [56]. Curcumin may modify the surface protein structure, thus inhibiting entry of virus and budding. It can also affect the protein membrane by altering the host cell’s lipid bilayer composition [57]. A new study showed that curcumin has a binding affinity to two proteins that are involved in the adhesion, fusion, and entry of the corona virus [57].

A flavanone from citrus fruit, Hesperidin, and its metabolite Hesperetin can suppress the entry of COVID-19 by blocking its ability to bind to the ACE2 cellular receptor, therefore reducing its protein expression [58].

Nutritional interventions during acute infections also involve prevention, diagnosis, or treatment of malnutrition in order to improve short- and long-term complications. Specifically, deficiencies in micronutrients are associated with adverse clinical outcomes during viral infections. It has been proposed that, other than the abovementioned micronutrients, vitamin A, B vitamins, omega-3 polyunsaturated fatty acids, and iron should be considered in the assessment of micronutrients among COVID-19 patients. Thus, provision of daily allowances for vitamins and trace elements is integral among malnourished patients [57].

The adopted dietary pattern can also affect the body’s inflammation level and immune response [59]. Pro-inflammatory diets that are characterized by excess intake of sugars, hydrogenated fat and chemical additives can manipulate the body’s response to infections by inducing the release of free radical and toxins. On the other hand, anti-inflammatory diets can protect the body from infections including respiratory infections and COVID-19. Such diets have high intakes of fruit, vegetables, and fish oil [60]. Among children, dietary habits have been closely associated with respiratory infections. A case–control study showed that frequent consumption of processed food (OR = 2.268, 95% confidence intervals 1.163–4.424), high-meat diet (OR = 1.830, 95% confidence intervals 1.358–2.467), and high-sugar diet (OR = 2.614, 95% confidence intervals 1.363–5.014) were positively associated with respiratory infection risk [61]. Additionally, a cohort of pregnant women in North America women exhibited an inversely dose-related relationship between high fruit and vegetable intake and the incidence of ARI (*p* = 0.03) [61]. A recent literature review by Van der Gaag showed that pre- and pro-biotic food (milk, garlic, kiwi, and fish oil) can prevent respiratory tract infections and even reduce the duration of anti-biotic dependence. However, the effectiveness of these foods is yet to be tested on COVID-19 [62].

## 5. Discussion

Despite the promising results of the presented interventions, their efficacy and safety warrant further investigation. Regarding the usage of CHM, one of the major concerns of the WHO regarding its usage is the lack of confirmed mechanisms proving both its efficacy and safety [63]. Moreover, CHM use is usually unsupervised, and thus there is no monitoring, and potential interactions or adverse effects in COVID-19 patients can be detected. Unsupervised CHM intake can also potentially result in unintentional intoxication or infection [24].

With vitamin D deficiency being a global health problem, supplements are being prescribed to adjust this status. However, vitamin D supplements should be prescribed on the basis of the circulating 25(OH) D. If administered irrespective of blood values, supplements can be regarded as unsafe and could induce hyperglycemia, morbidity and mortality [64]. Indeed, high bolus doses of vitamin D reduce its efficacy for non-classic effects while increasing adverse outcomes. The high efficacy of vitamin D supplements has only been detected among ARI patients with low baseline vitamin D status [65].

A review assessing both safety and efficacy of vitamin C in the management of ARI and COVID-19 showed that vitamin C does aid in relieving the symptoms and respiratory functions; however, to date, high-quality clinical trials are needed to test its safety, and whether it should be supplemented orally or intravenously [45]. COVID-19 patients are being supplemented with doses that are higher than recommended, and it still remains to be confirmed whether or not supplements only control the short-term health risks of the disease [65]. The safety of vitamin C supplements has been reported for doses up to 2 g/day, with possible adverse effects starting at levels of 3–4 g/day. High doses of oral vitamin C can cause diarrhea, whereas IV vitamin C can lead to kidney infection.

Zinc supplementation in high doses can raise several safety concerns, such as anosmia and copper deficiency. As for its efficacy, results are still pending, as ongoing trials are still in progress [66]. Several studies have shown that both zinc and vitamin C supplements in high doses show no benefits, suggesting a lack of efficacy [45].

Overall, additional long-term observational studies and RCTs are needed to obtain a more comprehensive insight into the effects of CHM and supplements on COVID-19. Among the CHM, the effects and action of YP, particularly, could be further investigated. It was not only shown to possess antiviral properties, but YP is the only CHM that is unanimously recommended by all six provinces in China for use in the fight against COVID-19. Regarding usage of nutritional supplementation to date, vitamin D has shown promising results in COVID-19 studies, and thus its role warrants more exploration [67].

## 6. Conclusions

In conclusion, many herbal and nutritional treatments play a role in the prevention and fight against respiratory infections, including COVID-19. Alternative therapy has shown promising results both in vitro and in vivo, as adjunct, or even as mono-therapy. Further clinical studies are needed to establish these alternative treatments as part of the management of emerging respiratory infections.

## Figures and Tables

**Table 1:** Completed and planned clinical trials on the effects of Chinese herbal medicine (CHM) in patients with COVID-19.

Reference	Objective	Intervention	Findings
Hu et al., 2021 [15]	To determine the efficacy and safety of LH capsules among COVID-19 patients	4 LH capsules daily compard to usual treatment	Higher recovery rate (*p* = 0.0022), clinical cure (*p* = 0.017), and lower recovery time from symptoms in treatment group
Liu et al., 2020 [18]	To evaluate the effectiveness and safety of Jinhua Qinggan granules as a treatment for COVID-19 patients	Jinhua Qinggan capsule upon admission compared to control (no capsule)	Seven day viral clearance rate was higher (*p* = 0.009) and the recovery time for pneumonia was shorter (*p* = 0.021) in treatment group
Zhang et al., 2020 [19]	To explore the effifacy of Jiaweidayuan as a treatment for COVID-19 patients	conventional treatment combined with Jiaweidayuan compared to conventional treatment alone	Improved overall clinical symptoms in treatment group such as CRP and lymphocytes (*p* < 0.05)
Ye, Y.A., 2020 [20]	To test the effectiveness of the classic CHM formula (*maxingshigantang*, *yinqiaosan*, *dayuanyin*, *xiaochaihutang*) as a treatment for severe COVID-19 patients	CHM with standard care or the standard care alone	Odds of a shift toward death were lower in CHM with standard care group (not significant)
Gao et al., 2021 [21]	To investigate how CHM *(Rehmanniae Radix*, *Rehmanniae Redix Praeparata*, *Ophiopogonis Radix*, *Lilii Bulbus*, *Paeoniae Radix Alba*, *Angelicae Sinensis Radix*, *Fritillariae Thunbergii Bulbus*, *Glycyrrhizae Radix et Rhizoma*, *Platycodonis Radix*, and *Salviae Miltiorrhizae Radix et Rhizoma)* can regulate immune function and autoimmune deficiency among COVID-19 patients and how exercise aids in their recovery	Patients randomized to either: - Cardiorespiratory exercise + CHM - CHM - Exercise	In progress
Andrew Shubov et al., 2021 [22]	To establish the safety and feasibility of the use of modified Qing Fei Pai Du Tang among COVID-19 patients	Modified Qing Fei Pai Du Tang (eight capsules three times a day) compared to placebo	In progress
